# R-Spondins Are Expressed by the Intestinal Stroma and are Differentially Regulated during *Citrobacter rodentium-* and DSS-Induced Colitis in Mice

**DOI:** 10.1371/journal.pone.0152859

**Published:** 2016-04-05

**Authors:** Eugene Kang, Mitra Yousefi, Samantha Gruenheid

**Affiliations:** Department of Microbiology and Immunology and Complex Traits Group, McGill University, Montreal, Quebec, Canada; Child & Family Research Institute, CANADA

## Abstract

The R-spondin family of proteins has recently been described as secreted enhancers of β-catenin activation through the canonical Wnt signaling pathway. We previously reported that *Rspo2* is a major determinant of susceptibility to *Citrobacter rodentium*-mediated colitis in mice and recent genome-wide association studies have revealed *RSPO3* as a candidate Crohn’s disease-specific inflammatory bowel disease susceptibility gene in humans. However, there is little information on the endogenous expression and cellular source of R-spondins in the colon at steady state and during intestinal inflammation. RNA sequencing and qRT-PCR were used to assess the expression of R-spondins at steady state and in two mouse models of colonic inflammation. The cellular source of R-spondins was assessed in specific colonic cell populations isolated by cell sorting. Data mining from publicly available datasets was used to assess the expression of R-spondins in the human colon. At steady state, colonic expression of *R-spondins* was found to be exclusive to non-epithelial CD45^-^ lamina propria cells, and *Rspo3/RSPO3* was the most highly expressed R-spondin in both mouse and human colon. R-spondin expression was found to be highly dynamic and differentially regulated during *C*. *rodentium* infection and dextran sodium sulfate (DSS) colitis, with notably high levels of *Rspo3* expression during DSS colitis, and high levels of *Rspo2* expression during *C*. *rodentium* infection, specifically in susceptible mice. Our data are consistent with the hypothesis that in the colon, R-spondins are expressed by subepithelial stromal cells, and that *Rspo3/RSPO3* is the family member most implicated in colonic homeostasis. The differential regulation of the R-spondins in different models of intestinal inflammation indicate they respond to specific pathogenic and inflammatory signals that differ in the two models and provides further evidence that this family of proteins plays a key role in linking intestinal inflammation and homeostasis.

## Introduction

Consisting of four members (R-spondin1-4), the R-spondin family of secreted proteins has recently emerged as potent enhancers of canonical Wnt signaling [1]. All four R-spondin family members likely share similar biological activities as evidenced by structure and functional analyses: R-spondins bind the stem cell receptors Lgr4-6 and the transmembrane E3 ubiquitin ligases Znrf3/Rnf43 to potentiate Wnt signaling by modulating Wnt receptor turnover [2–7]. Although they have roles in many tissues, R-spondins are of particular importance in the gastrointestinal tract where they have been shown to be crucial for the maintenance of intestinal stem cells [8]. These stem cells at the base of intestinal crypts mediate the vigorous self-renewal of the intestinal epithelium and give rise to transit amplifying cells which divide before they terminally differentiate into specialized cell types such as enterocytes and goblet cells [9]. Mature cells then undergo apoptosis a few days after their terminal differentiation and are shed into the gut lumen.

The canonical Wnt/β-catenin signaling pathway plays a major role in regulating epithelial cell fate and represents the first driving force behind the proliferation of intestinal epithelial precursors [10]. Genetic manipulations have demonstrated the critical role of this pathway in intestinal homeostasis and the fatal consequences of either too much or too little Wnt signaling. Conditional ablation of β-catenin from the intestinal epithelium blocks proliferation of epithelial precursors leading to crypt degeneration and loss, intestinal failure, and death [11]. Conversely, mutations in *APC*, a component of the β-catenin destruction complex, result in hyperproliferation of intestinal crypts and the loss of fully differentiated cells, also leading to death [12]. These data highlight the critical need for balanced Wnt signaling to ensure intestinal homeostasis.

Like Wnt signaling itself, emerging evidence indicates that R-spondin activity must also be kept in a fine balance to maintain intestinal health. Activating translocations of *RSPO2* and *RSPO3* that are mutually exclusive with other Wnt-activating mutations have been shown to drive the development of colon cancer [13–14], and we recently demonstrated through a forward genetics approach that *Rspo2* is a major determinant of susceptibility to *C*. *rodentium*-mediated infectious colitis in mice [15]. Susceptible mice (e.g. C3H/HeOuJ) share a unique genetic haplotype immediately upstream of *Rspo2*, driving high levels of *Rspo2* in susceptible mouse strains during infection and leading to pathological activation of Wnt signaling, loss of intestinal differentiation, and animal death [15–17]. Inhibition of R-spondin-mediated pathways by recombinant Dkk1 administration improved outcome in these susceptible mice [15]. In contrast, C3H/HeOuJ mice carrying a congenic segment encompassing *Rspo2* and its regulatory region from resistant mice (C3Ou.B6-*Cri1*) do not upregulate *Rspo2* during infection, and instead suffer from self-limiting disease with no mortality [15]. Conversely, studies in mice have shown that exogenous R-spondin1 treatment can promote the recovery of intestinal stem cells after radiation-induced damage [18] and be beneficial in several experimental colitis models including DSS-induced colitis by stimulating crypt cell growth and promoting intestinal healing [19]. Furthermore, meta-analysis of genome-wide association studies (GWAS) of inflammatory bowel disease (IBD) identified a SNP within *RSPO3* as a Crohn’s disease-specific susceptibility locus (rs9491697, p = 3.79E-10, OR = 1.08) [20]. However, as with the majority of loci identified in GWAS studies, the causal variant underlying this association has not been identified, and it is not known whether a gain- or loss- of function at *RSPO3* could be implicated in Crohn’s disease susceptibility.

Taken together, these reports indicate that R-spondins may link intestinal inflammation and homeostasis, but also identify an urgent need to better understand the roles of endogenous R-spondins in healthy and inflamed intestinal tissue, for which there is currently little information. While several groups have studied the effects of treating the intestinal epithelium with exogenous R-spondins, few have examined the endogenous expression of R-spondins in the gut. This work focuses on assessing expression levels of *Rspo1-4* in the colon at steady state and during intestinal inflammation using two IBD-relevant mouse models in which R-spondins were shown to be highly regulated.

## Materials and Methods

### Ethics Statement

All breeding and experimental procedures were conducted in strict accordance with the Canadian Council of Animal Care and approved by the McGill University Animal Care Committee (permit #5009). Mice were euthanized by CO_2_ asphyxiation and all efforts were made to minimize suffering.

### *In vivo C*. *rodentium* infection

C3H/HeOuJ (henceforth called C3Ou) (Jackson Laboratory, Bar Harbor, ME) and C3Ou.B6-*Cri1* congenic mice [17] with an introgressed segment of chromosome 15 (entitled *Cri-1*) from C57BL/6 mice on the C3Ou genomic background were maintained in a specific-pathogen free animal facility at McGill University and provided standard mouse chow and water *ad libitum*. *C*. *rodentium* strain DBS100 was grown overnight in 3 ml of Luria-Bertani (LB) medium shaking at 37°C. Five-week-old mice were infected by oral gavage with 0.1 ml of LB medium containing 2–3 x 10^8^ colony-forming units of bacteria. The infectious dose was confirmed by plating of serial dilutions. Mice were monitored daily and euthanized on experimental days 3, 6, and 9 and their distal colons were dissected and snap frozen in liquid nitrogen for RNA isolation.

### DSS-induced colitis and histology

To assess the role of R-spondins during DSS colitis and during a post-DSS repair period, colitis was induced in 7-week-old male mice by adding 3% (w/v) DSS (MP Biomedicals) at 36–50 kDa to the drinking water for 6 days before returning to normal drinking water for 9 days. Mice were then euthanized on select time points and their colon sections were collected for histology and RNA isolation. Body weight was measured every other day. For histology, colon sections were fixed in 10% buffered formalin, paraffin-embedded, sectioned at 5 μm, and stained for hematoxylin and eosin (H&E). H&E sections were scanned on the ScanScope XT digital scanner (Leica) and images were obtained using the ImageScope software (Leica).

### RNA sequencing

Total RNA of whole colon tissues from uninfected and infected C3Ou and C3Ou.B6-*Cri1* mice was isolated using TRIzol (Invitrogen) according to the manufacturer’s instructions. A cleanup of the samples was done using the RNeasy Plus Micro Kit (Qiagen). The RNA integrity number, assessed by a Bioanalyzer (Agilent), was 8.0 and above for all RNA samples. Sequencing was performed at the McGill University and Genome Quebec Innovation Center using Illumina HiSeq 2000/2500 technology with three libraries per lane to generate 110–187 million paired reads per library. The data was aligned to the mm10 mouse genome assembly (http://genome.ucsc.edu/cgi-bin/hgGateway?db=mm10) with the combination of the TopHat/Bowtie software [21]. The Cufflinks program [22] was used to calculate the relative abundance of select transcripts of interest, expressed in FPKM (“fragments per kilobase of exon per million fragments mapped”) units. Human gene expression data were acquired from the Human Protein Atlas (http://www.proteinatlas.org/), available through the ArrayExpress Archive (http://www.ebi.ac.uk/arrayexpress/) under the accession number E-MTAB-2836.

### Quantitative real-time polymerase chain reaction (qRT-PCR)

For the *C*. *rodentium*-mediated colitis model, total RNA from colons was isolated using TRIzol according to the manufacturer’s instructions. For the DSS-mediated colitis model, total RNA from colons was isolated using the ToTALLY RNA system (Ambion) with the lithium chloride precipitation step to remove all traces of DSS and gross DNA contamination. The purity of RNA was assessed by a spectrophotometer; all samples had a 260/280 absorbance ratio between 1.8 and 2.0. Complementary DNA was synthesized from 1 μg of RNA with ProtoScript II reverse transcriptase (NEB) and random primers (Invitrogen) using an Eppendorf PCR thermal cycler. Expression levels of *Rspo1-4*, *EpCAM* and *Ptprc* were measured using TaqMan Gene Expression Assays (Applied Biosystems) and expression levels of *Mmp7* and *c-Myc* were measured using SYBR Green PCR Master Mix (Life Technologies) on the Applied Biosystems StepOnePlus Real-Time PCR system. Analysis was performed according to the comparative C^T^ method using *Hprt* as the housekeeping gene. The primer pairs for SYBR Green assays are as follows: *Mmp7* forward: GCATTTCCTTGAGGTTGTCC, *Mmp7* reverse: CACATCAGTGGGAACAGGC, *c-Myc* forward: TGACCTAACTCGAGGAGGAGCTGGAATC, *c-Myc* reverse: AAGTTTGAGGCAGTTAAAATTATGGCTGAAGC, *Hprt* forward: GTTGGATACAGGCCAGACTTTGTTG, *Hprt* reverse: GATTCAACTTGCGCTCATCTTAGGC.

### Cell sorting

Colonic epithelial and lamina propria cells from mice were isolated using a modified version of a previously described method [23]. In brief, colons were collected, cut open longitudinally into 1 cm pieces, and washed in calcium- and magnesium-free HBSS (Gibco) supplemented with 2% heat-inactivated fetal calf serum (FCS, Wisent) and 15 mM HEPES (Gibco). The resulting tissue pieces were washed in calcium- and magnesium-free HBSS supplemented with 2% FCS, 15 mM HEPES, and 5 mM EDTA to remove epithelial cells, which were then collected by centrifugation. After removing the supernatant, the tissue pieces were incubated in RPMI-1640 (Sigma) supplemented with 10% FCS, 15 mM HEPES, 160 μg/ml collagenase IV (Sigma) and 40 μg/ml DNAse I (Roche) for 40 min at 37°C. The cell suspension was filtered through a 70 μm cell strainer (Sigma) before proceeding with antibody staining. Cells were stained with viability dye (Life Technologies) and surface antibody CD45.2 (eBioscience) and sorted on the FACSAria II (BD Biosciences) into CD45^+^ (hematopoietic) and CD45^-^ (non-hematopoietic) populations. R-spondin expression was assessed by qRT-PCR using *Gapdh* as the housekeeping gene.

### Data Analysis

Data analyses were performed using GraphPad Prism v6.0 software. Gene expression data were analyzed by the Mann-Whitney test with p values <0.05 being considered significant.

## Results

### Relative expression of *R-spondin* genes in the normal uninflamed colon

**Fig 1:**
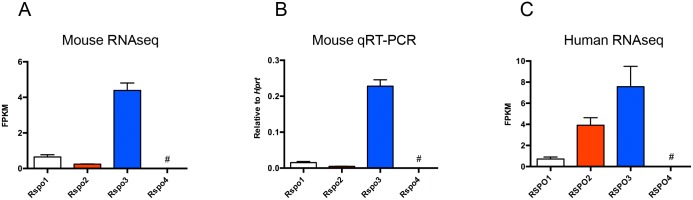
Relative expression of *R-spondin* genes in the normal colon of mice and humans. (A) R-spondin levels in C3Ou mice colon at steady state as acquired by RNAseq (n = 3). (B) Expression of *Rspo1-4* by qRT-PCR in colonic samples from C3Ou mice normalized to *Hprt* (n = 6). Graph is representative of both C3Ou and C3Ou.B6-*Cri1* mice. (C) Expression data from normal human colon were analyzed from the publicly available ArrayExpress Archive under the accession number E-MTAB-2836 (n = 7). Error bars represent mean ±SEM. # = undetected.

RNA sequencing of whole colon tissues from C3Ou mice was performed to investigate the gene expression of *Rspo1-4* at steady state (Fig 1A). *Rspo3* expression was relatively high while *Rspo1* and *Rspo2* had a similar, low expression pattern. *Rspo4* was not detected by RNA sequencing. To investigate whether the genetic haplotype at *Rspo2* had any effect on overall *Rspo* expression, we also performed RNA sequencing on whole colons of C3Ou.B6-*Cri1* congenic mice bearing the resistance locus at *Rspo2* and found *Rspo* expression levels to be indistinguishable from that of C3Ou mice (S1 Fig).

To confirm these results and to validate the use of TaqMan-based qRT-PCR for our subsequent studies, we also assessed the expression of *Rspo1-4* by qRT-PCR in colonic samples from susceptible C3Ou and resistant C3Ou.B6-*Cri1* congenic mice. Consistent with RNA sequencing, we found the *Rspo3* gene to be expressed at high levels at steady state and the hierarchy of expression to be *Rspo3*>*Rspo1*>*Rspo2*>*Rspo4* with *Rspo4* under the limit of detection (Fig 1B).

In order to assess R-spondin expression in human colon, we mined online databases of RNAseq-derived gene expression provided by the Human Protein Atlas (Fig 1C). Similar to what was found in mouse colon, this dataset showed *RSPO3* as being expressed at the highest level followed by lower levels of *RSPO2* and *RSPO1*. The *RSPO2/Rspo2* expression level was found to be higher in the human dataset compared to our mouse studies (4 vs 1 FPKM). Like in our mice studies, *RSPO4* was not detected in these samples.

### R-spondins are expressed by intestinal stromal cells

The cellular source(s) of R-spondins in the gut is an unanswered question that hinders our understanding of their pathophysiological roles in intestinal health and disease. Our published *in situ* hybridization, immunohistochemistry, and bone marrow chimera experiments support the hypothesis that colonic *Rspo2* is expressed in radio-resistant sub-epithelial stromal cells [15]. This has been independently confirmed by several studies indicating the stromal compartment as a source of R-spondins [24–26].

**Fig 2:**
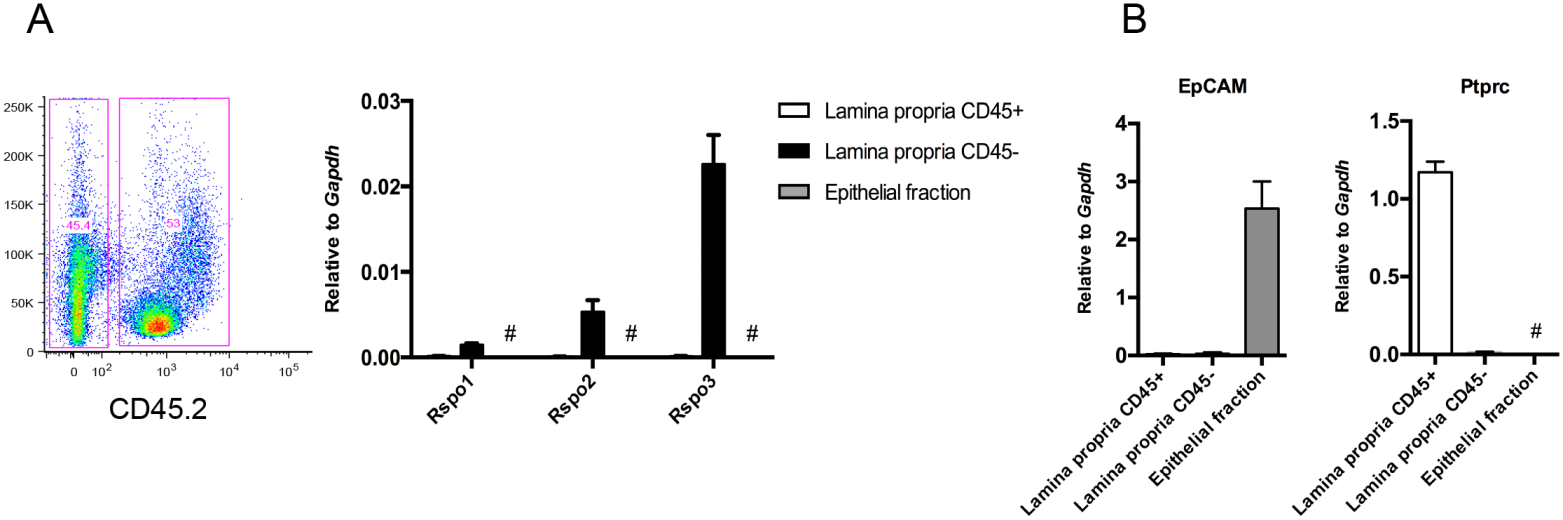
R-spondins are expressed by sub-epithelial non-hematopoietic stromal cells. Colonic epithelial and lamina propria cells from C3Ou mice were isolated, and lamina propria cells were further stained for CD45.2 and sorted into CD45^+^ and CD45^-^ populations after gating on singlet and live cells. R-spondin (A), *EpCAM* and *Ptprc* (B) expression was measured by qRT-PCR and normalized to *Gapdh* (n = 3). Error bars represent mean ±SEM. # = undetected.

To systematically characterize the R-spondin-expressing cell populations in the normal colon, we isolated colonic epithelial and lamina propria cells from mice and further sorted the lamina propria cells into CD45^+^ and CD45^-^ populations. Following RNA isolation and cDNA preparation, *Rspo1-3* expression was determined using TaqMan-based qRT-PCR. Analysis of the lamina propria cell populations revealed that *Rspo1-3* mRNA transcripts were expressed exclusively in CD45^-^ (non-hematopoietic) cells and were nearly undetectable in the CD45^+^ (hematopoietic) cells (Fig 2A). Epithelial cells did not express detectable levels of any of the R-spondins, which is consistent with publicly available microarray data showing higher enrichment of *Rspo1-3* in the mesenchymal fraction compared to the epithelial fraction of the perinatal mouse intestine [27]. As a measure of quality control, *EpCAM* mRNA transcript levels were assessed to rule out any contamination of epithelial cells in the hematopoietic and non-hematopoietic stromal populations (Fig 2B). *Ptprc* mRNA transcript levels were also assessed to confirm CD45 expression specifically in the hematopoietic population.

### Modulation of R-spondin expression during *C*. *rodentium*–mediated colitis

*C*. *rodentium*-mediated infectious colitis is a widely recognized model for studying intestinal inflammation. Our results outlined above indicate that *Rspo3* is the major R-spondin expressed in the colon. Moreover, since *RSPO3* is a candidate locus for susceptibility to IBD, and since exogenous R-spondin1 administration was previously shown to be beneficial in some mouse colitis models, we examined the expression of all four *R-spondin* genes during *C*. *rodentium*-mediated colitis in susceptible C3Ou mice.

**Fig 3:**
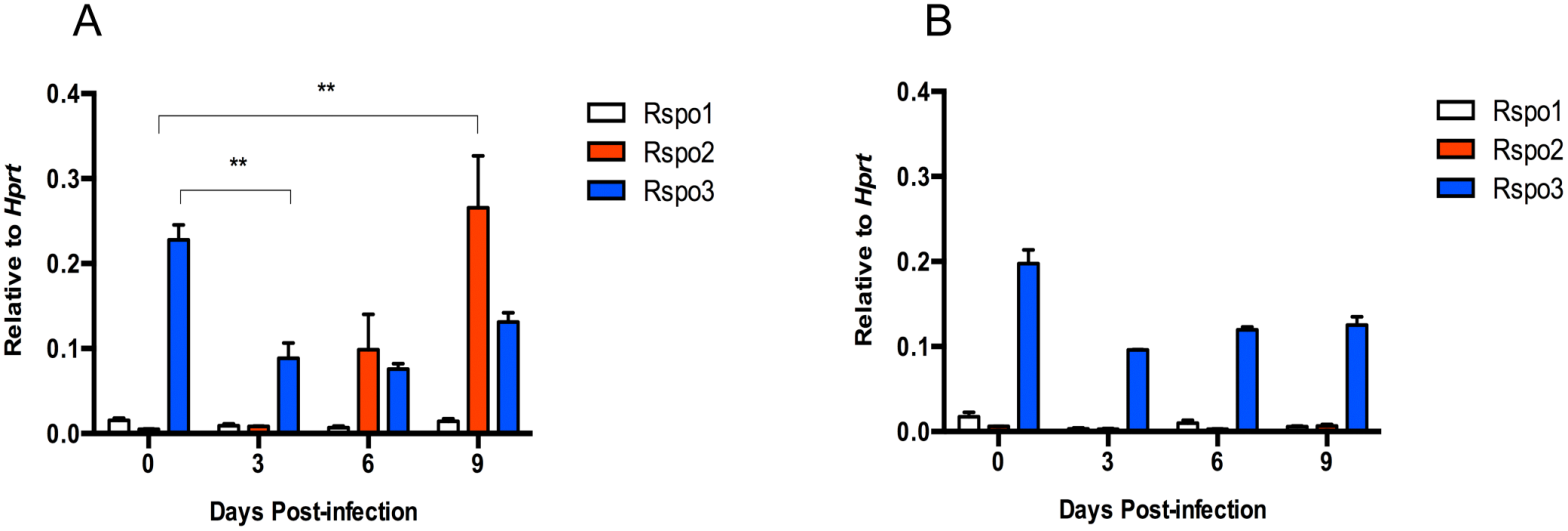
Modulation of R-spondin expression during *C*. *rodentium*–mediated colitis. Mice were infected by oral gavage (2–3 x 10^8^ CFU of bacteria) and euthanized on experimental days 3, 6, and 9. Relative quantification of *Rspo1-4* expression in C3Ou (A) (n = 6 per time point) and C3Ou.B6-*Cri1* (B) (n = 3 per time point) mice was assessed by qRT-PCR and normalized to *Hprt*. *Rspo4* was not detected. **p<0.01. Error bars represent mean ±SEM.

Expression of *Rspo1-4* was measured by qRT-PCR in colonic samples from mice that were left uninfected or at days 3, 6, and 9 post-infection. Consistent with our previous data, we found the *Rspo2* gene to be strongly and continuously induced during infection. In contrast, *Rspo3* expression was downregulated 2-fold by day 3 of infection while *Rspo1* was downregulated 2-fold by day 6 of infection (Fig 3A). *Rspo4* was under the limit of detection. To determine if the downregulation of *Rspo1* and *Rspo3* was potentially a compensatory response to the increase in *Rspo2* during infection, we assessed R-spondin levels in resistant C3Ou.B6-*Cri1* congenic mice at days 3, 6, and 9 post-infection. R-spondin1-4 expression in resistant congenic mice was found to mirror that of susceptible mice with the exception that there was no significant upregulation of *Rspo2* mRNA transcripts (Fig 3B). Since we were unable to detect *Rspo4* at steady state or throughout the course of infection, we did not pursue *Rspo4* in any further experiments.

### R-spondin expression levels are regulated during DSS colitis

**Fig 4:**
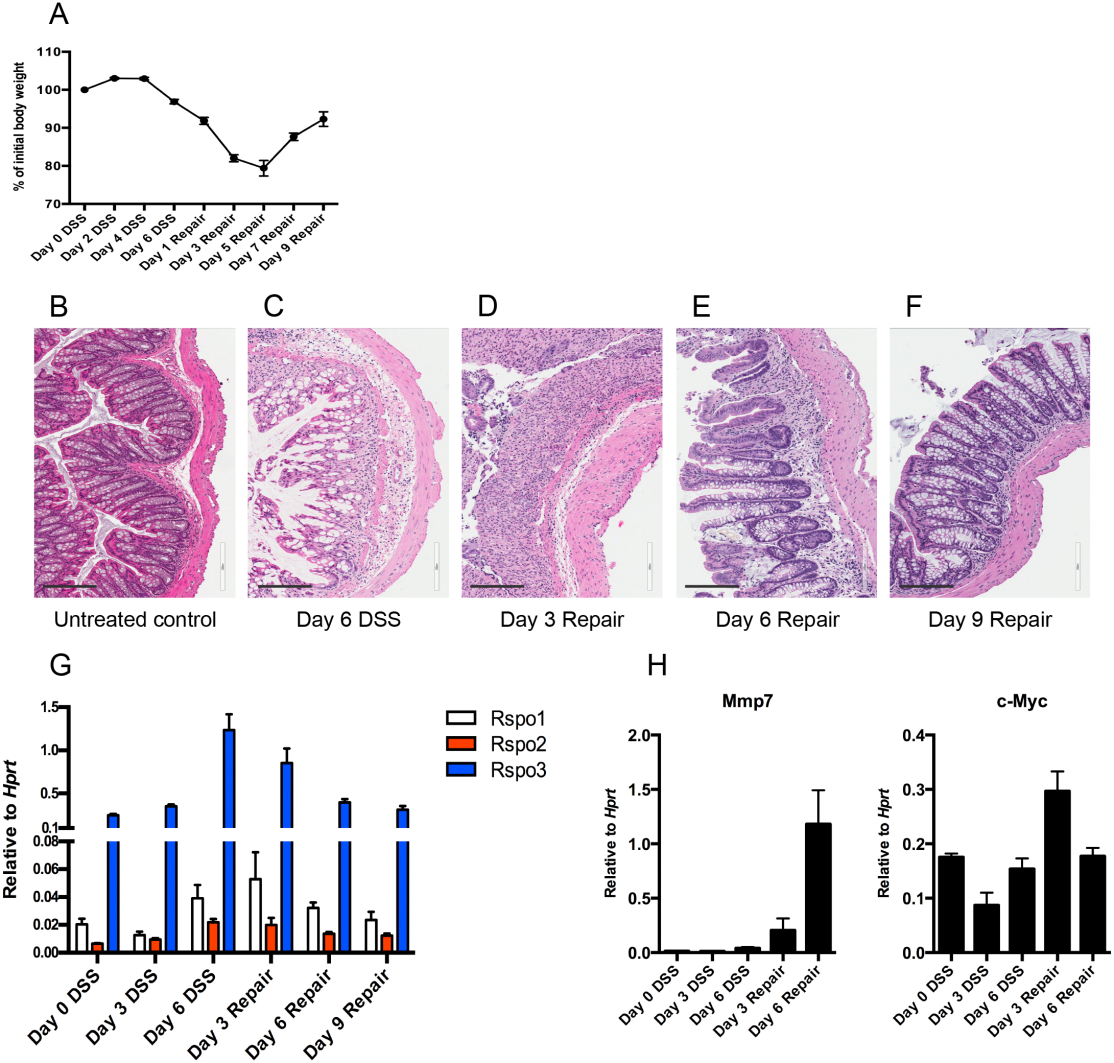
R-spondin expression levels are regulated during tissue repair following DSS injury. C3Ou mice were subjected to 3% DSS administration for 6 days before returning to normal drinking water for 9 days. Body weights (A) were measured every other day during the course of DSS treatment and during repair (n = 4–20 per time point, aggregate of two experiments). Histological changes were examined on untreated controls (B), at day 6 of DSS (C), and on days 3 (D), 6 (E), and 9 (F) following DSS removal at 15X magnification. Scale bars, 200 μm. *Rspo1-3* levels were measured by qRT-PCR on days 0, 3, and 6 of DSS and on days 3, 6, and 9 post-DSS (G) (n = 4 per time point). Wnt target genes *Mmp7* and *c-Myc* were measured by qRT-PCR on days 3 and 6 of DSS and on days 3 and 6 post-DSS and were compared to untreated controls (H) (n = 3 per time point). Error bars represent mean ±SEM.

To assess R-spondin modulation in an additional model of intestinal inflammation, we induced colitis in mice using DSS to examine the expression of R-spondins during the acute phase of colitis and subsequent repair period following DSS withdrawal. C3Ou mice were administered 3% DSS for 6 days before returning to normal drinking water for 9 days. Mice continued to lose body weight after DSS withdrawal until day 5 post-DSS when mice began to gradually re-gain their body weight (Fig 4A). Histological changes in the colon were examined on day 6 of DSS and on days 3, 6, and 9 of repair. Whereas untreated control sections showed intact epithelium with well-defined crypts (Fig 4B), DSS-treated mice had sub-mucosal edema, immune cell infiltration, epithelial disruption and loss of crypts by day 6 of DSS treatment (Fig 4C). Similar changes were observed 3 days after DSS withdrawal with additional areas of erosion and loss of entire crypts (Fig 4D). Evidence of intestinal repair including attenuation of lesions and regeneration of crypts was evident by day 6 of DSS withdrawal (Fig 4E) followed by near full recovery of the mucosa by day 9 of withdrawal (Fig 4F).

**Fig 5:**
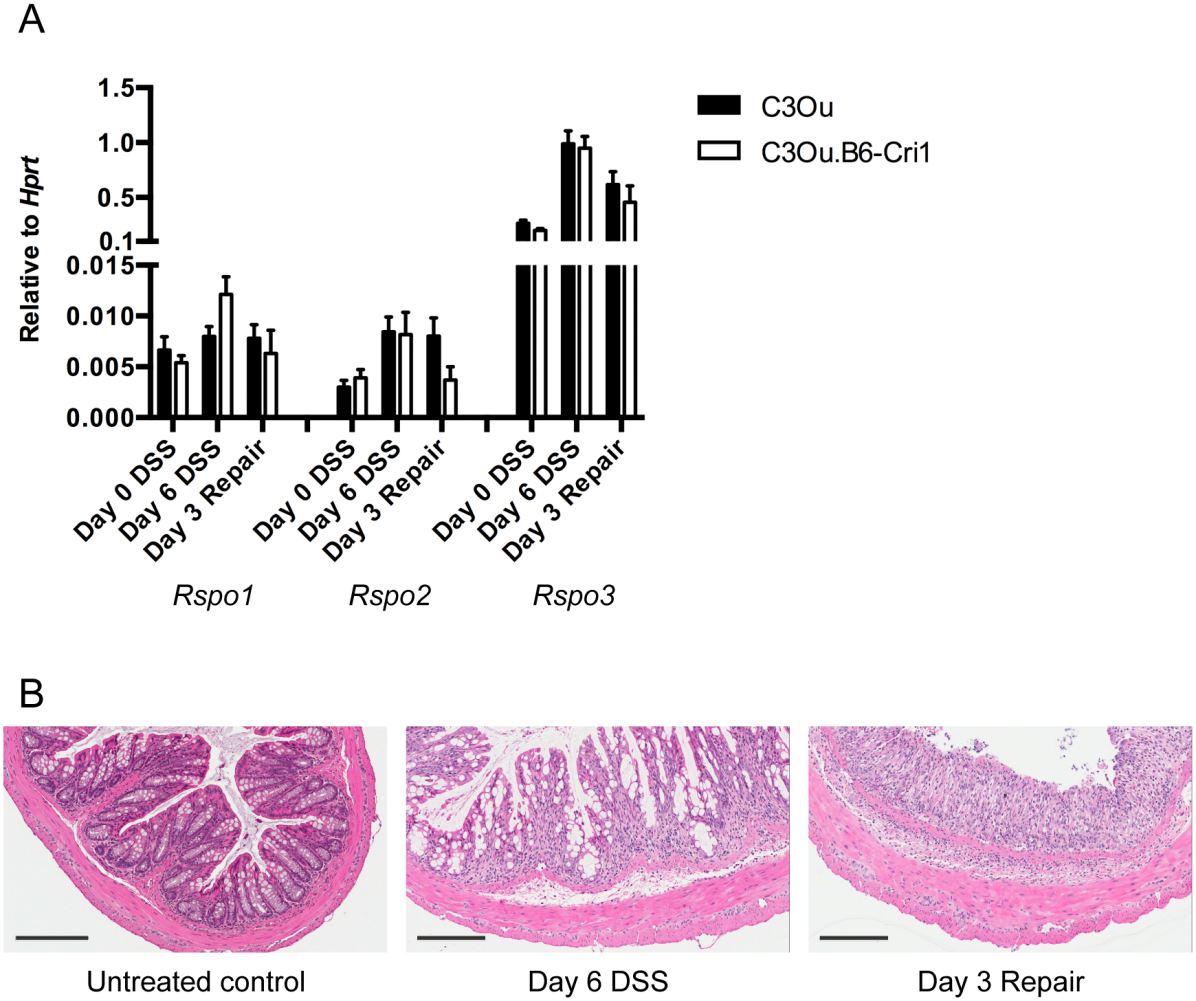
R-spondin expression is similar in C3Ou and C3Ou.B6-*Cri1* mice during DSS treatment and repair. C3Ou.B6-*Cri1* mice were administered 3% DSS for 6 days before returning to normal drinking water for 3 days. Relative quantification of *Rspo1-3* mRNA (A) and histological comparisons (B) were measured on day 6 of DSS and on day 3 of DSS withdrawal and were compared to untreated controls (n = 4–7 per time point, aggregate of 2 experiments). Error bars represent mean ±SEM. Scale bars, 200 μm.

Consistent with histological features, R-spondin mRNA levels peaked at day 6 of DSS and day 3 of withdrawal when colon sections showed the most damage with *Rspo1* induced 2-fold, *Rspo2* 3-fold, and *Rspo3* 5-fold when compared to untreated controls (Fig 4G). With already high expression at steady state relative to the other R-spondins, the 5-fold induction of *Rspo3* during DSS treatment resulted in markedly elevated absolute levels of this gene in the colon. Expression of all R-spondins returned to homeostatic levels by day 9 of recovery from DSS administration, suggesting an acute role for R-spondins in intestinal repair at the time points in which the epithelium is most damaged. This is perhaps not surprising given that Wnt signaling is activated during intestinal regeneration [28]. Indeed, we found the Wnt target genes *Mmp7* and *c-Myc* to be induced during the recovery phase following DSS withdrawal (Fig 4H). Notably, the induction of *Rspo2* expression was very modest in the DSS model as opposed to what was observed during *C*. *rodentium* infection (3-fold vs 50-fold). To examine if the *Rspo2* haplotype had any effect on *Rspo* expression in this model, R-spondin levels were subsequently measured in C3Ou.B6-*Cri1* congenic mice at day 6 of DSS and day 3 of withdrawal based on these two time points expressing the highest R-spondins in C3Ou mice. We did not observe any statistically significant differences in *Rspo1-3* expression between the two mouse strains (Fig 5A) or increased epithelial proliferation/repair in C3Ou mice as crypt architecture was similar to C3Ou.B6-*Cri1* congenic mice (Fig 5B). Together, these results highlight *Rspo3* as the dominant R-spondin in DSS colitis and provide evidence that *Rspo2* and *Rspo3* may respond to specific pathogenic and inflammatory signals that differ between the two colonic inflammation models.

## Discussion

Emerging evidence supports a central role for R-spondins—particularly *Rspo2* and *Rspo3* –in intestinal health and disease. Indeed, recurrent activating *RSPO2* and *RSPO3* gene fusions were found to occur in 10% of human colon tumors [13] and a recent follow-up study targeting *RSPO3* in a *RSPO3*-fusion tumor xenograft model was shown to downregulate genes expressed in the stem cell compartment and inhibit tumor growth [29]. These observations demonstrate the potential clinical relevance of targeting R-spondins in the treatment of colorectal tumors, and correspondingly an anti-*RSPO3* antibody [30] developed to target the R-spondin pathway is currently in phase I clinical trial. Additionally, meta-analysis of genome-wide association studies linked *RSPO3* with Crohn’s disease [20]. However, since it is not known whether a gain- or loss- of function at *RSPO3* is implicated in Crohn’s disease susceptibility, we can only speculate that a gain-of-function would promote Wnt-dependent intestinal proliferation but with the potentially harmful effect of limiting differentiation while a loss-of-function could limit intestinal repair. Taken together, these reports suggest that R-spondins may have a broad relevance in inflammation-associated intestinal diseases and warrant further investigation into the role of endogenous R-spondins in healthy and inflamed intestinal tissue, for which there is currently little information.

Our RNA sequencing and qRT-PCR analyses combined with publicly available human expression data revealed the *Rspo3*/*RSPO3* gene to be the dominant R-spondin expressed in the normal uninflamed colon, indicating that *Rspo3*/*RSPO3* is a major contributor to the potentiation of canonical Wnt signaling at steady state. However, despite their importance in embryonic development and tissue homeostasis the source of these secreted proteins is still poorly understood. Mice with targeted inactivation of the *Rspo2* gene die immediately after birth due to multiple organ defects [31–34], and likewise targeted disruption of the *Rspo3* gene leads to early embryonic lethality at around embryonic day 10 [35–36]. This prevents the assessment of R-spondin function in the intestine during postnatal development and disease conditions. Consistent with previous work, our gene expression analyses of isolated epithelial cells and sorted CD45^+^ and CD45^-^ colonic lamina propria cells restricted *Rspo1-3* expression to the CD45^-^ population. Future work will need to sort the R-spondin-expressing pool into further sub-populations using various hematopoietic and non-hematopoietic stromal cell markers specific for each of the different populations of mesenchymal cells that are present in the lamina propria. Our progress towards identifying the cell type expressing R-spondins can guide in the development of a conditional knockout mouse line to study the role of R-spondins specifically in the intestine without confounding effects from their roles in other tissues. In addition, it will provide the starting point for *in vitro* and *ex-vivo* analyses of which inflammatory mediators including IBD-relevant cytokines are important for R-spondin induction.

We have previously shown that pathological induction of *Rspo2* during *C*. *rodentium* infection leads to intestinal dysfunction and death in genetically susceptible mice [15]. We expanded on this study to examine the expression of all four *R-spondin* genes during *C*. *rodentium-*mediated colitis and DSS-induced colitis including a post-DSS repair period. R-spondins were found to be highly modulated during inflammation, with notably robust upregulation of *Rspo2* expression during *C*. *rodentium* infection in susceptible mice and upregulation of *Rspo3* expression during DSS colitis. The finding that *Rspo3* was the most highly induced R-spondin during DSS treatment with significantly elevated absolute expression levels of this gene highlights *Rspo3* as a potentially important mediator of Wnt signaling in the gut. In our DSS repair model, R-spondin levels continuously increased until crypt morphology gradually began to recover after several days following DSS withdrawal. This suggests a role for R-spondins in facilitating epithelial repair as a response to mucosal injury, which is consistent with enhancement of Wnt signaling during intestinal regeneration [28] and with the observations that genetic reduction or pharmacological inhibition of Dkk1 during DSS colitis has been shown to promote wound repair by increasing proliferation of epithelial cells [37].

The discovery that R-spondins are differentially regulated during enteric infection and DSS administration provides a novel avenue of investigation into the mechanisms of R-spondin gene regulation. Despite both models inducing intestinal inflammation, the difference in host response to enteric infection and chemical DSS may be responsible for the differential expression of the R-spondins; it may be that they require specific pathogenic and inflammatory signaling necessary for induction. Indeed, *C*. *rodentium* infection induces a robust Th1/Th17 response with increased gene expression of IFN-γ, interleukin-12 (IL-12), IL-17, and IL-22 [38–39] while acute DSS colitis activates a predominant Th1 response but with upregulation of several Th2 cytokines including IL-10 [40]. Elucidating this difference in host response may provide important insights in the mechanisms governing R-spondin expression in the intestinal tract.

In summary, our work exploited the *C*. *rodentium* infectious colitis model and the DSS colitis/repair model to explore R-spondin expression at steady state and during inflammation in the colon. We have shown that R-spondins are expressed by sub-epithelial non-hematopoietic stromal cells and that their expression is differentially and strongly regulated during *C*. *rodentium* infection and DSS colitis. Our data suggest that R-spondin-mediated signaling can be modulated by infectious or inflammatory stimuli and provides further evidence that this family of proteins plays a key role in linking intestinal inflammation and homeostasis.

## Supporting Information

S1 Fig Relative expression of *R-spondin* genes in the normal colon of C3Ou.B6-*Cri1* mice R-spondin levels in C3Ou.B6-*Cri1* mice colon at steady state as acquired by RNAseq (n = 3). Error bars represent mean ±SEM. # = undetected.
(TIF)
