# Supplementary figures and images for: R-Spondins Are Expressed by the Intestinal Stroma and are Differentially Regulated during Citrobacter rodentium- and DSS-Induced Colitis in Mice
Source: PLoS One. 2016 Apr 5;11(4):e0152859. doi: 10.1371/journal.pone.0152859 (PMC4821485; doi:10.1371/journal.pone.0152859)

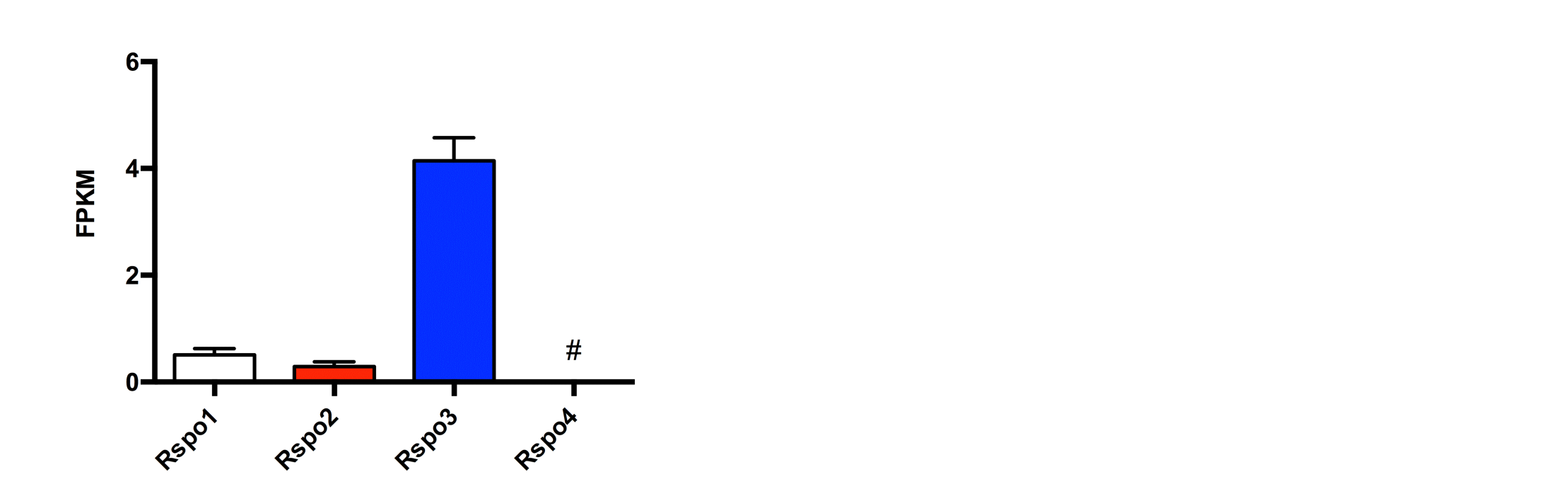

Supplement: S1 Fig — Error bars represent mean ±SEM. # = undetected. (TIF) [file pone.0152859.s001.tif]
